# Detection of endogenous S1292 LRRK2 autophosphorylation in mouse tissue as a readout for kinase activity

**DOI:** 10.1038/s41531-018-0049-1

**Published:** 2018-04-19

**Authors:** Jillian H. Kluss, Melissa M. Conti, Alice Kaganovich, Aleksandra Beilina, Heather L. Melrose, Mark R. Cookson, Adamantios Mamais

**Affiliations:** 1 Cell Biology and Gene Expression Section, Laboratory of Neurogenetics, National Institute on Aging, National Institutes of Health, Bethesda, MD USA; 2 Department of Neuroscience, Mayo Clinic, 4500 San Pablo Rd S, Jacksonville, FL 32224 USA

## Abstract

Parkinson’s disease-linked mutations in *LRRK2* enhance the kinase activity of the protein, therefore targeting LRRK2 kinase activity is a promising therapeutic approach. Phosphorylation at S935 of LRRK2 and of its Rab GTPase substrates have proven very useful biomarkers to monitor its kinase activity. Complementary to these approaches autophosphorylation of LRRK2 can be used as a direct kinase activity readout but to date detection of autophosphorylation at endogenous levels in vivo has been limited. We developed a fractionation-based enrichment method to successfully detect endogenous S1292 LRRK2 autophosphorylation in mouse tissues and highlight S1292 as a physiological readout candidate for LRRK2 kinase activity in vivo.

## Introduction

Parkinson’s disease (PD) is a prevalent age-dependent neurodegenerative movement disorder for which there currently is no cure or biomarker available. Mutations in several genes segregate with PD in families, including *LRRK2* that codes for the kinase Leucine-rich repeat kinase 2.^1^ The G2019S substitution in the kinase domain of LRRK2 confers increased kinase activity and has been linked to neuronal toxicity.^2–4^ Thus, targeting the kinase activity of LRRK2 has been proposed as a therapeutic strategy for PD and a number of tool compounds have been developed. Reliable methods to monitor drug efficacy are required for the clinical application of targeting LRRK2. Recently, a subset of Rab GTPases including Rab8a and Rab10 were identified as key physiological substrates of LRRK2.^5^ Detection of LRRK2-mediated phosphorylation of these substrates has been successful at endogenous levels in tissue and in human peripheral blood neutrophils and peripheral blood mononuclear cells and was shown to respond to pharmacological kinase inhibition.^6–8^ These studies nominate detection of Rab GTPase phosphorylation as a promising method of monitoring LRRK2 inhibition in the clinic. In parallel to this approach monitoring LRRK2 phosphorylation can also be used as a readout of its kinase activity.

LRRK2 is constitutively phosphorylated at S935 among other sites and different pathways have been reported to mediate these phosphorylation events.^9,10^ While S935 is not an autophosphorylation site, it responds to LRRK2 kinase inhibition^11^ and has been used extensively as an indirect marker of activity in cells and tissue. Recently, S935 dephosphorylation was validated as a pharmacodynamic biomarker in PD patients^12^ and is currently been used as a readout in a phase-I trial of LRRK2 kinase inhibitors (Denali Therapeutics). While S935 is a very useful tool, phosphorylation of this site does not reflect LRRK2 activity in some instances. S935 phosphorylation is decreased by the PD-linked mutations R1441C and Y1699C,^13^ while these pathogenic variants show increased kinase activity toward Rab GTPases.^5^

The autophosphorylation site S1292 has been proposed as a physiological and direct marker of LRRK2 kinase activity. Kinase inhibition has been shown to induce S1292 dephosphorylation in vivo and in vitro, several PD-linked mutations enhance this phosphorylation, and kinase-dead LRRK2 is not phosphorylated at this residue.^14^ However, to date, detection of pS1292 has only been possible in models of LRRK2 overexpression in mice^14^ or in urine after exosome enrichment in humans^15^ but not at endogenous levels in tissue. To address this problem, we developed a protocol to enrich LRRK2 and detect endogenous S1292 phosphorylation in vivo. We showed that through a fractionation-based enrichment method we can robustly detect LRRK2 S1292 phosphorylation and validated this by genetic means, using G2019S LRRK2 knock-in and knock-out mice, and pharmacological approaches, via in vivo administration of the LRRK2-kinase inhibitor MLi-2.

## Results

**Fig. 1:**
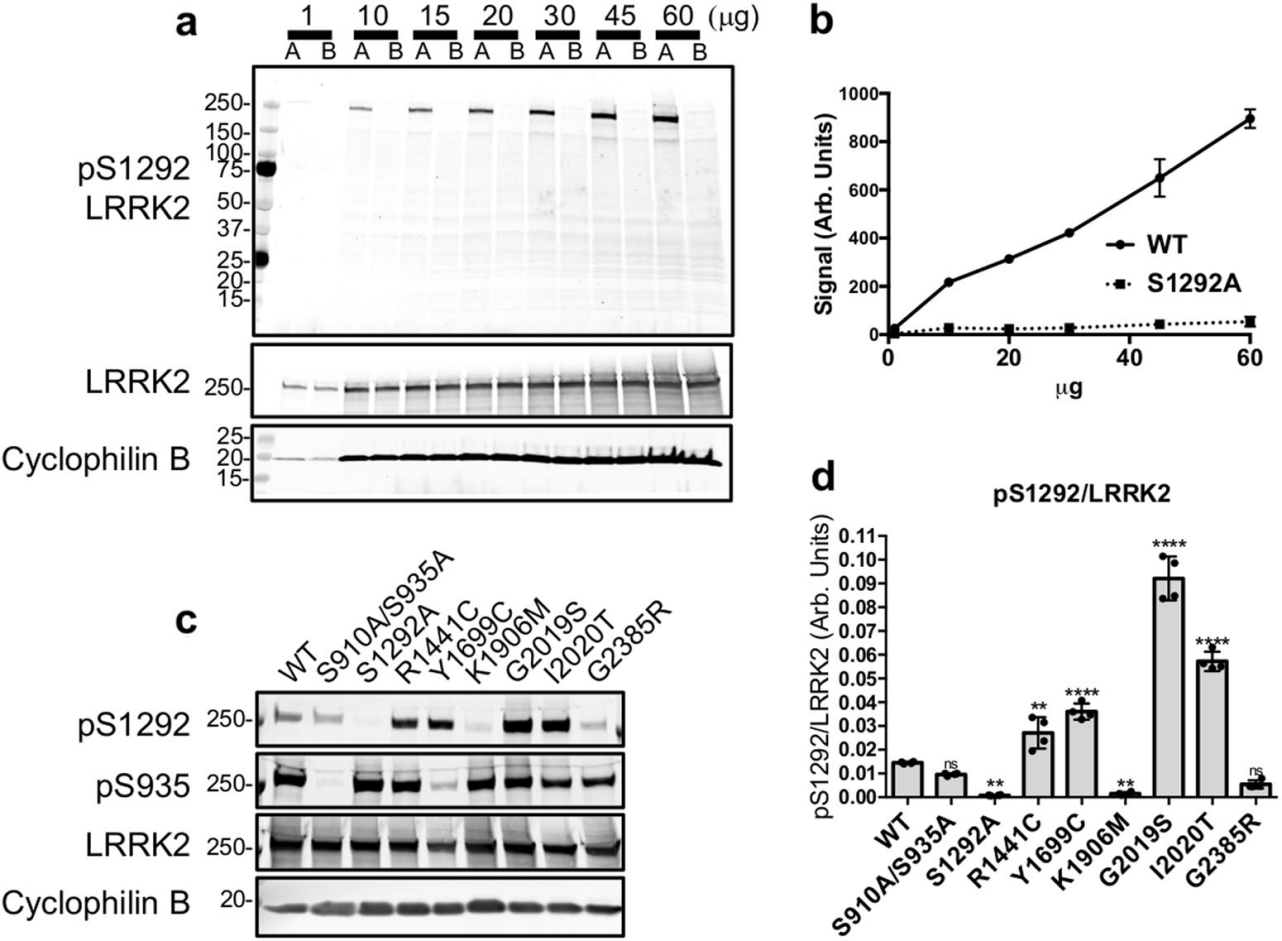
Characterization of pS1292 LRRK2 antibody. **a** Increasing amounts of HEK293FT lysates transiently expressing WT LRRK2 (A) or S1292A LRRK2 (B) were analyzed by western blot and probed with the commercially available anti-pS1292 LRRK2 antibody (ab203181). The antibody showed minimal non-specific bands and linear detection in the range tested (**b**) (*n* = 3 blots; mean with SD bars shown). Transiently expressed LRRK2 genetic variants were probed for S1292 phosphorylation (**c**, **d**). Strong pS1292 signal was detected in the G2019S and I2020T kinase hyperactive variants and no signal detected at the S1292A phospho-null and K1906M kinase-dead variant. The R1441C and Y1699C variants showed increased S1292 phosphorylation compared to WT which is in accordance to previous literature (**c**, **d**) (*n* = 4; one-way ANOVA with Tukey’s post hoc test; *F*(8, 27) = 210.1)

We first characterized the specificity of a commercially available anti-pS1292 LRRK2 antibody (Fig. 1). Wild type (WT) or S1292A LRRK2 were transiently expressed in HEK293FT cells and increasing amounts of lysates were analyzed by western blot. The antibody detected WT LRRK2 with a linear response between 10 and 40 µg of lysate but only background signal was noted with S1292A LRRK2 (Fig. 1a, b). To confirm that PD-associated LRRK2 mutations increase S1292 phosphorylation,^14^ HEK293FT cells were transiently transfected with WT, S910A/S935A, S1292A, R1441C, Y1699C, K1906M, G2019S, I2020T, and G2385R LRRK2 constructs and blotted for total and phosphorylated LRRK2 antibodies (Fig. 1c). Consistent with published data,^14^ R1441C, G2019S, and I2020T were found to increase S1292 phosphorylation compared to WT, while the G2385R mutation had pS1292 levels similar to WT (Fig. 1d). Interestingly, the Y1699C variant also showed higher pS1292 compared to WT similar to prior reports.^16,17^ The kinase-dead K1906M variant showed no S1292 phosphorylation.

**Fig. 2:**
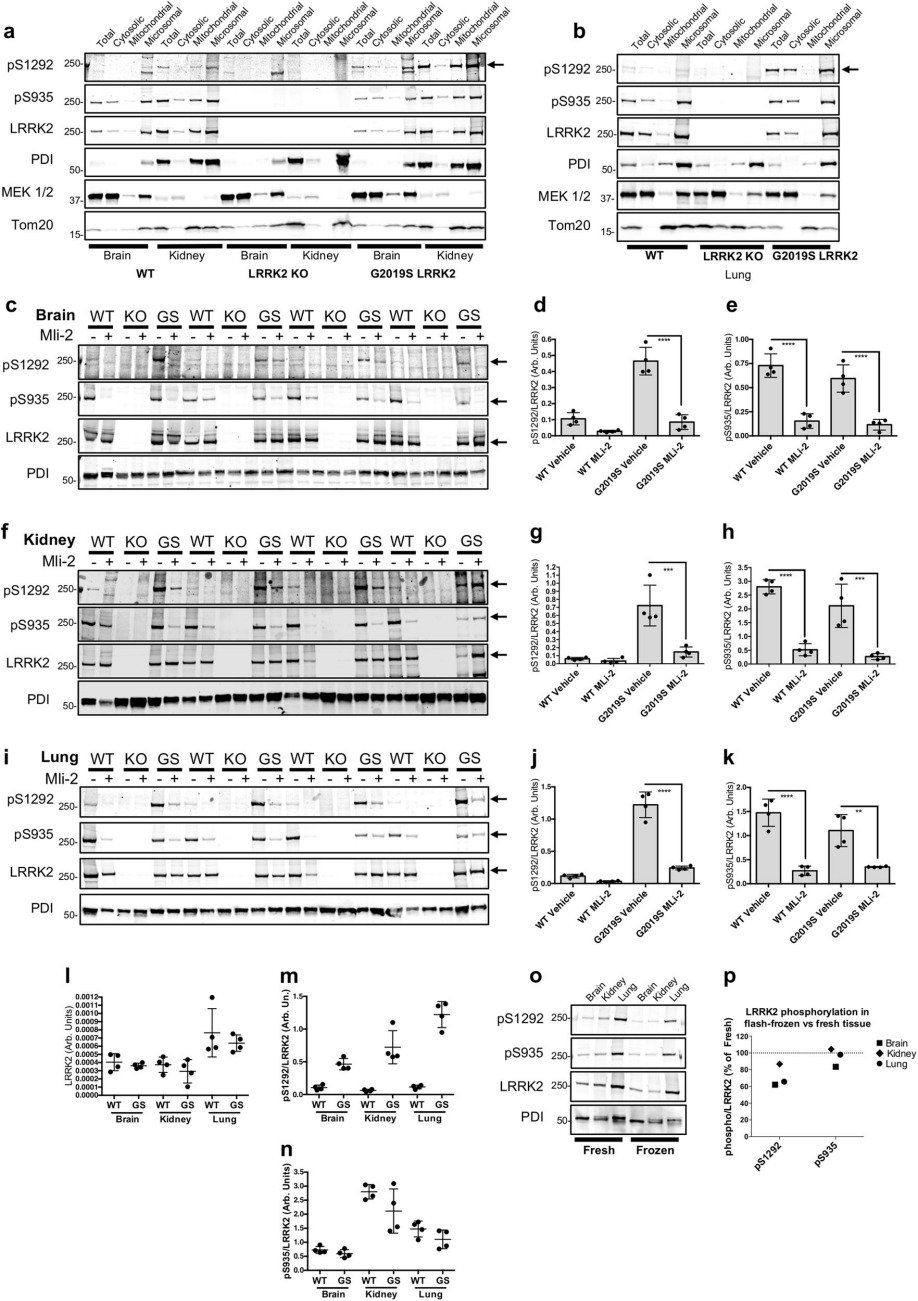
Genetic and pharmacological validation of pS1292 LRRK2 detection in mouse tissue. Brain, kidney, and lung mouse tissue were fractionated and total, cytosolic, crude mitochondrial, and crude microsomal fractions were analyzed by western blot (**a**, **b**). S1292 phosphorylation was detectable in WT tissue and microsomal enrichment enhanced detection in brain and kidney. Tissue from G2019S knock-in mice showed higher S1292 phosphorylation to WT and Lrrk2 knock-out mice were used to validate the real band. Antibodies against PDI, MEK 1/2, and Tom20 were used to detect markers for microsomes, cytosolic, and mitochondrial fractions, respectively (**a**, **b**). WT, Lrrk2 KO, and G2019S (GS) mice were treated with the LRRK2 kinase inhibitor MLi-2 before tissue was collected and processed (**c**, **f**, **i**). Analysis of microsomal enriched fractions showed higher S1292 LRRK2 in G2019S compared to WT tissue. Kinase inhibition induced a significant decrease in S1292 phosphorylation in all G2019S tissues examined (**d**, **g**, **j**). Both WT and G2019S showed strong signal for S935 phosphorylation that was ameliorated by MLi-2 (**e**, **h**, **k**). Quantitation of phosphorylation levels is presented as S1292 or S935 phosphorylation over total LRRK2 levels (****P < 0.0001, ***P < 0.0003, **P = 0.0022; *n* = 4; mean with SD bars shown; one-way ANOVA with Tukey’s post hoc; **d** *F*(3, 12) = 57.27, **e** *F*(3, 12) = 34.92, **g** *F*(3, 12) = 24.31, **h** *F*(3, 12) = 32.29, **j** *F*(3, 12) = 118.4, **k** *F*(3, 12) = 27.53)). Data from **c**, **f**, and **i** vehicle-treated mice were plotted to compare total and phosphorylated LRRK2 levels across tissues (**l**, **m**, **n**). Total LRRK2 was normalized to input protein amounts of the ultracentrifugation step (see Methods). Higher LRRK2 levels were observed in lung compared to brain and kidney (**l**) (two-way ANOVA; tissue, *P* = 0.0002; genotype, *P* = 0.1954; *n* = 4; SD bars shown). Higher pS1292 was seen in lung compared to brain and kidney for G2019S tissue (two-way ANOVA; tissue, *P* < 0.0001; genotype, *P* < 0.0001; *n* = 4; SD bars shown), while brain and lung showed lower pS935 compared to kidney in WT and G2019 tissue (two-way ANOVA; tissue, *P* < 0.0001; genotype, *P* = 0.0222; *n* = 4; SD bars shown). S1292 and S935 phosphorylation were analyzed in fresh-processed and flash-frozen tissue from a G2019S LRRK2 mouse (**o**, **p**). Phospho/total LRRK2 values were plotted as the percentage of phosphorylation of flash-frozen over fresh-processed tissue (**o**, **p**)

A major challenge in using pS1292 to monitor LRRK2 activity of endogenous protein is the apparent low stoichiometry of this modification. Previous studies have described detection of S1292 phosphorylation in brain lysates from BAC transgenic mice that overexpress LRRK2^14^ but there are no reports of pS1292 detection in animals at physiological levels of LRRK2. To address this problem we optimized detection of endogenous S1292 phosphorylation in vivo using a subcellular fractionation method previously used in our lab to examine LRRK2 localization showing enrichment of LRRK2 in the crude microsomal fraction.^18,19^ Here, we fractionated brain, kidney, and lung mouse tissue and analyzed total, cytosolic, crude mitochondrial, and crude microsomal fractions by western blot. We used Lrrk2 knockout mice to confirm identity of the protein bands and LRRK2 G2019S knock-in mice^20^ to test for ability to detect enhanced kinase activity. We detected some pS1292 in the total fractions but signal was enriched in the microsomal fractions allowing detection across tissues (Fig. 2a, b). G2019S knock-in showed consistently higher S1292 phosphorylation compared to WT tissue and there was no band at ~250 kDa in Lrrk2 knock-out tissue. These results show that the use of microsomal fractionation allowed us to analyze endogenous tissue and consistently detect S1292 autophosphorylation in multiple mouse tissues.

To validate the fractionation protocol we tested for the microsomal marker PDI and show enrichment in the crude microsomal fraction (Fig. 2a, b). We do, however, detect some PDI in the mitochondrial fraction predominantly in kidney and carryover of Tom20 between the mitochondrial and microsomal fractions. Looking more closely at different markers we observed carryover of the outer mitochondrial marker VDAC1 to the microsomal fraction but minimal carryover of COX IV, an inner mitochondrial membrane marker (data not shown). We conclude that this protocol has possible limitations across different tissues in terms of purity of microsomes but successfully achieves LRRK2 enrichment and detection of low-stoichiometry phosphorylation.

We additionally validated our detection of endogenous pS1292 LRRK2 in vivo using the LRRK2 kinase inhibitor MLi-2.^21,22^ We found that acute MLi-2 administration induces dephosphorylation of both S1292 and S935 (Fig. 2c–k). The decrease in pS1292 phosphorylation in WT tissue after treatment did not reach statistical significance, possibly due to the very low levels of phosphorylation that are close to the detection limit. However, the G2019S tissues gave consistently higher S1292 phosphorylation levels compared to the WT and these were significantly diminished after treatment with MLi-2 (Fig. 2d, g, j). The levels of S935 phosphorylation responded to MLi-2 in WT and G2019S mice to a comparable degree (Fig. 2e, h, k). These results demonstrate that both pS1292 and pS935 respond to kinase inhibition across tissues at the endogenous level in vivo. A normalizer sample was included in all gels of Fig. 2c, f, and i that allowed us to compare total and phosphorylated LRRK2 levels across multiple tissues. Higher LRRK2 levels were detected in lung vs. brain and kidney in enriched microsomes when normalized to starting protein amounts (Fig. 2l). pS1292 levels in G2019S mice were higher in lung vs. brain and kidney (Fig. 2m), while pS935 was higher in kidney compared to brain and lung in both WT and G2019S mice. This suggests that LRRK2 phosphorylation events may be differentially regulated across different tissues.

While all the tissue examined here was processed fresh as a precaution, flash-freezing of tissue for later analysis is a standard procedure. To test whether our method precludes using frozen tissue, we investigated the effect of flash-freezing on LRRK2 phosphorylation. Tissue was collected from a G2019S LRRK2 mouse (4 months old) and the brain was separated into left and right hemispheres, lung tissue was split in half and the kidneys were processed separately. Half the tissue was flash-frozen on dry-ice, while the other half was processed fresh for microsomal fractionation. The frozen tissue was defrosted on ice before being processed. Upon analysis we observed a slight decrease in pS1292 LRRK2 in frozen tissue compared to freshly processed, while S935 phosphorylation was not affected (Fig. 2o, p). This suggests that while microsomal enrichment can be used for S1292 LRRK2 detection in flash-frozen tissue, some loss of phosphorylation may occur. This might become a problem when looking at WT LRRK2 that has lower stoichiometry of S1292 phosphorylation compared to the kinase hyperactive G2019S.

## Discussion

Methods to monitor LRRK2 kinase activity are useful in the development of therapeutic interventions that target this protein. Recently, a subset of Rab GTPases have been reported as physiological substrates of LRRK2, and detection of their phosphorylation has been nominated as a readout of LRRK2 kinase activity in patient-derived material.^6–8^ This opens new avenues in monitoring pathway-specific LRRK2 activity and is a promising tool in the clinic and in research. Rab GTPases are vesicular proteins with highly specialized functions imposed in part by the different subcellular compartments they reside in, suggesting that their phosphorylation by LRRK2 is under distinct control mechanisms that are poorly understood. Monitoring LRRK2 autophosphorylation in addition to Rab GTPase phosphorylation can serve as a global LRRK2 activity readout and a complementary in vivo approach. In the current study, we present a method to detect the autophosphorylation of LRRK2 in vivo at the endogenous level. Using a fractionation-based enrichment method we detected endogenous S1292 LRRK2 phosphorylation in different mouse biomatrices and validated this by genetic means, using knock-in and knock-out models, and pharmacological means, by in vivo kinase inhibition. To establish a clinical relevance, it will be important to validate this technique in patient-derived material. Reliable detection of phosphorylated LRRK2 will be useful in developing clinical biomarkers of PD in at least two contexts. First, augmented LRRK2 kinase activity and autophosphorylation may serve as biomarkers for presymptomatic diagnosis of PD, as autophosphorylation state has been correlated with PD.^15^ Second, pS1292 LRRK2 could be used to monitor the efficacy of pharmacological kinase inhibition in a therapeutic context. It is of interest in the latter context that, at least for MLi-2, there was a correlation between the level of loss of pS1292 in brain compared to peripheral tissues, suggesting that monitoring pS1292 LRRK2 outside of the brain might be predictive of target engagement in the CNS. While our data suggest that both pS935 and pS1292 respond to kinase inhibition in vivo, pS1292 dephosphorylation is a reliable readout in G2019S but not WT mouse tissue (Fig. 2d, g, j). Thus, pS935 may be a better readout when looking at WT tissue and this may be taken into consideration if LRRK2 inhibitors are to be administered to idiopathic PD patients. The correlation of LRRK2 autophosphorylation and Rab GTPase phosphorylation in the context of response to in vivo LRRK2 inhibition merits further investigation for their application as biomarkers in the clinic.

## Methods

### FLAG LRRK2 constructs

FLAG-tagged (3×) WT LRRK2 was cloned in a pCHMWS plasmid (generous gift from Dr. Jean-Marc Taymans, Jean-Pierre Aubert Research Center, France) and mutations were introduced using a QuikChange II XL Site-Directed Mutagenesis Kit (Stratagene) as previously described.^9^

### Animal procedures

Animal procedures were performed in accordance with a protocol approved by the Institutional Animal Care and Use Committee of the National Institute on Aging, NIH. WT, Lrrk2 KO, or homozygous G2019S LRRK2 C57BL/6J mice (8–10 months old) were randomized for genotype, treatment, and sex using the sample function in R. Mice were injected subcutaneously with vehicle [40% (w/v) Hydroxypropyl-β-Cyclodextran] or MLi-2 (3 mg/kg dissolved in vehicle) and euthanized 1 h after treatment.^5^ *N* = 4 mice per genotype per treatment group were used. Based on a previous publication reporting pS935 dephosphorylation following MLi-2 administration in mice,^22^ we estimated that *N* = 4 gives >80% power to reject the null hypothesis at alpha = 0.05. Based on our data presented here, *N* = 4 achieves 90% power to detect a difference of effect size 6 at alpha = 0.05, in S1292 dephosphorylation in G2019S LRRK2 brain following acute MLi-2 treatment. No blinding method was used during the analysis of these data.

### Enrichment by fractionation

Fresh mouse brain, kidney, and lungs were homogenized on ice in 15% (w/v) sedimentation buffer [3 mM Tris/HCl pH 7.4, 250 mM sucrose, 0.5 mM EGTA, phosphatase inhibitors (Thermo Fisher Scientific HALT phosphatase inhibitor cocktail 100×), and protease inhibitors (ROCHE cOmplete)]. Homogenates were centrifuged at 3000 × *g* for 10 min at 4 °C, and the supernatants from this spin were centrifuged at 5000 × *g* for 10 min at 4 °C. This extra step of low-speed centrifugation was found to minimize carryover of undissolved material from the pellet. The supernatant from this step was termed cleared homogenate. 200 μl of cleared homogenate were supplemented with 1× Cell Signaling Lysis buffer (Cat #9803), 0.1% SDS, and 0.5% sodium deoxycholate, kept on ice for an hour to complete lysis and centrifuged for 10 min at 23,000 × *g* at 4 °C. The supernatants from this step were run as the total fraction.

The remainder of cleared homogenates were centrifuged at 12,000 × *g* for 10 min at 4 °C, and the pellets (crude mitochondrial fraction) were resuspended in resuspension buffer at volume equal to the original sample [resuspension buffer: sedimentation buffer supplemented with 1× Cell Signaling Lysis buffer (Cat #9803), 0.1% SDS, 0.5% sodium deoxycholate. Final concentrations: 23 mM Tris-HCl, pH 7.4, 150 mM NaCl, 250 mM sucrose, 1% Triton X-100, 0.1% SDS, 0.5% sodium deoxycholate, 1 mM EDTA, 1.5 mM EGTA, 2.5 mM sodium pyrophosphate, 1 mM β-glycerophosphate, 1 mM Na3VO4, 1 µg/ml leupeptin, phosphatase inhibitors (Thermo HALT), and protease inhibitors (cOmplete)]. From the supernatants, 2 mg for brain, 2.5 mg for kidney, and 3 mg for lung were centrifuged at 100,000 × *g* for 1 h at 4 °C and the pellets were kept as microsomal fractions, while the supernatants were saved as cytosolic fractions. The microsomal fractions were resuspended by sonication on ice (3 × 15 s) in 80 µl of resuspension buffer.

### Western blotting

Samples were supplemented with NuPage LDS sample buffer 4× (NP0008), boiled for 5 min at 95 °C and 5 µl were run on a Bio-Rad Criterion™ TGX™ polyacrylamide gel (Cat #5671095) at 200 V for 37 min. Gels were transferred to nitrocellulose (Cat #1704159) on a Bio-Rad Trans-Blot^®^ Turbo™ transfer system at 20 V for 10 min. The nitrocellulose was blocked for 1 h in 50% TBS (20 mM Tris, 0.5 M NaCl, pH 7.5), 50% Odyssey blocking buffer (Li-Cor; 927–40,000) and incubated overnight with primary antibodies in 50% TBS-T (20 mM Tris, 0.5 M NaCl, pH 7.5, 0.1% Tween 20), 50% Odyssey blocking buffer at 4 °C. Following 3 × 5 min washes with TBS-T, the nitrocellulose was incubated at RT with secondary antibodies for 1 h, washed 3 × 5 min and scanned at the Li-Cor platform.

All primary antibodies were used at 1:2000 dilution: pS1292 LRRK2 (MJF-19-7-8; ab203181), total LRRK2 (ab133474), pS935 LRRK2 (ab133450), cyclophilin B (ab16045), PDI (Cell Signaling; 2446), MEK1/2 (Cell Signaling; 9122), and Tom20 (sc-11415). Secondary antibodies were used at 1:15,000 dilution: IRDye^®^ 800CW Goat anti-Rabbit IgG (LiCor: 926–32211). All blots presented in each figure panel were derived from the same experiment and were processed in parallel.

### Data availability

All data generated or analyzed during this study are included in this published article.

## Electronic supplementary material

Supplementary Figure

## References

[1] Hernandez DG, Reed X, Singleton AB (2016). Genetics in Parkinson disease: Mendelian vs. non-Mendelian inheritance. J. Neurochem..

[2] Greggio E (2006). Kinase activity is required for the toxic effects of mutant LRRK2/dardarin. Neurobiol. Dis..

[3] West AB (2007). Parkinson’s disease-associated mutations in LRRK2 link enhanced GTP-binding and kinase activities to neuronal toxicity. Hum. Mol. Genet..

[4] Dusonchet J (2011). A rat model of progressive nigral neurodegeneration induced by the Parkinson’s disease-associated G2019S mutation in LRRK2. J. Neurosci..

[5] Steger M (2016). Phosphoproteomics reveals that Parkinson’s disease kinase LRRK2 regulates a subset of Rab GTPases. eLife.

[6] Fan Y (2018). Interrogating Parkinson’s disease LRRK2 kinase pathway activity by assessing Rab10 phosphorylation in human neutrophils. Biochem. J..

[7] Lis P (2018). Development of phospho-specific Rab protein antibodies to monitor in vivo activity of the LRRK2 Parkinson’s disease kinase. Biochem. J..

[8] Thirstrup K (2017). Selective LRRK2 kinase inhibition reduces phosphorylation of endogenous Rab10 and Rab12 in human peripheral mononuclear blood cells. Sci. Rep..

[9] Chia R (2014). Phosphorylation of LRRK2 by casein kinase 1α regulates *trans*-Golgi clustering via differential interaction with ARHGEF7. Nat. Commun..

[10] Dzamko N (2012). The IkappaB kinase family phosphorylates the Parkinson’s disease kinase LRRK2 at Ser935 and Ser910 during Toll-like receptor signaling. PLoS ONE.

[11] Deng X (2011). Characterization of a selective inhibitor of the Parkinson’s disease kinase LRRK2. Nat. Chem. Biol..

[12] Perera G, Ranola M, Rowe DB, Halliday GM, Dzamko N (2016). Inhibitor treatment of peripheral mononuclear cells from Parkinson’s disease patients further validates LRRK2 dephosphorylation as a pharmacodynamic biomarker. Sci. Rep..

[13] Nichols RJ (2010). 14-3-3 binding to LRRK2 is disrupted by multiple Parkinson’s disease-associated mutations and regulates cytoplasmic localization. Biochem. J..

[14] Sheng Z (2012). Ser1292 autophosphorylation is an indicator of LRRK2 kinase activity and contributes to the cellular effects of PD mutations. Sci. Transl. Med..

[15] Fraser KB, Moehle MS, Alcalay RN, West AB (2016). Urinary LRRK2 phosphorylation predicts parkinsonian phenotypes in G2019S LRRK2 carriers. Neurology.

[16] Purlyte E (2018). Rab29 activation of the Parkinson’s disease‐associated LRRK2 kinase. EMBO J..

[17] Reynolds A, Doggett EA, Riddle SM, Lebakken CS, Nichols RJ (2014). LRRK2 kinase activity and biology are not uniformly predicted by its autophosphorylation and cellular phosphorylation site status. Front. Mol. Neurosci..

[18] Rudenko IN (2012). The G2385R variant of leucine-rich repeat kinase 2 associated with Parkinson’s disease is a partial loss of function mutation. Biochem. J..

[19] Beilina A (2014). Unbiased screen for interactors of leucine-rich repeat kinase 2 supports a common pathway for sporadic and familial Parkinson disease. Proc. Natl Acad. Sci. USA.

[20] Yue M (2015). Progressive dopaminergic alterations and mitochondrial abnormalities in LRRK2 G2019S knock in mice. Neurobiol. Dis..

[21] Scott JD (2017). Discovery of a 3-(4-pyrimidinyl) indazole (MLi-2), an orally available and selective leucine-rich repeat kinase 2 (LRRK2) inhibitor that reduces brain kinase activity. J. Med. Chem..

[22] Fell MJ (2015). MLi-2, a potent, selective, and centrally active compound for exploring the therapeutic potential and safety of LRRK2 kinase inhibition. J. Pharmacol. Exp. Ther..

