# Supplementary material for: Detection of endogenous S1292 LRRK2 autophosphorylation in mouse tissue as a readout for kinase activity
Source: NPJ Parkinsons Dis. 2018 Apr 19;4:13. doi: 10.1038/s41531-018-0049-1 (PMC5908918; doi:10.1038/s41531-018-0049-1)
Supplement: Supplementary file 1 — Supplementary Figure [file 41531_2018_49_MOESM1_ESM.pdf]

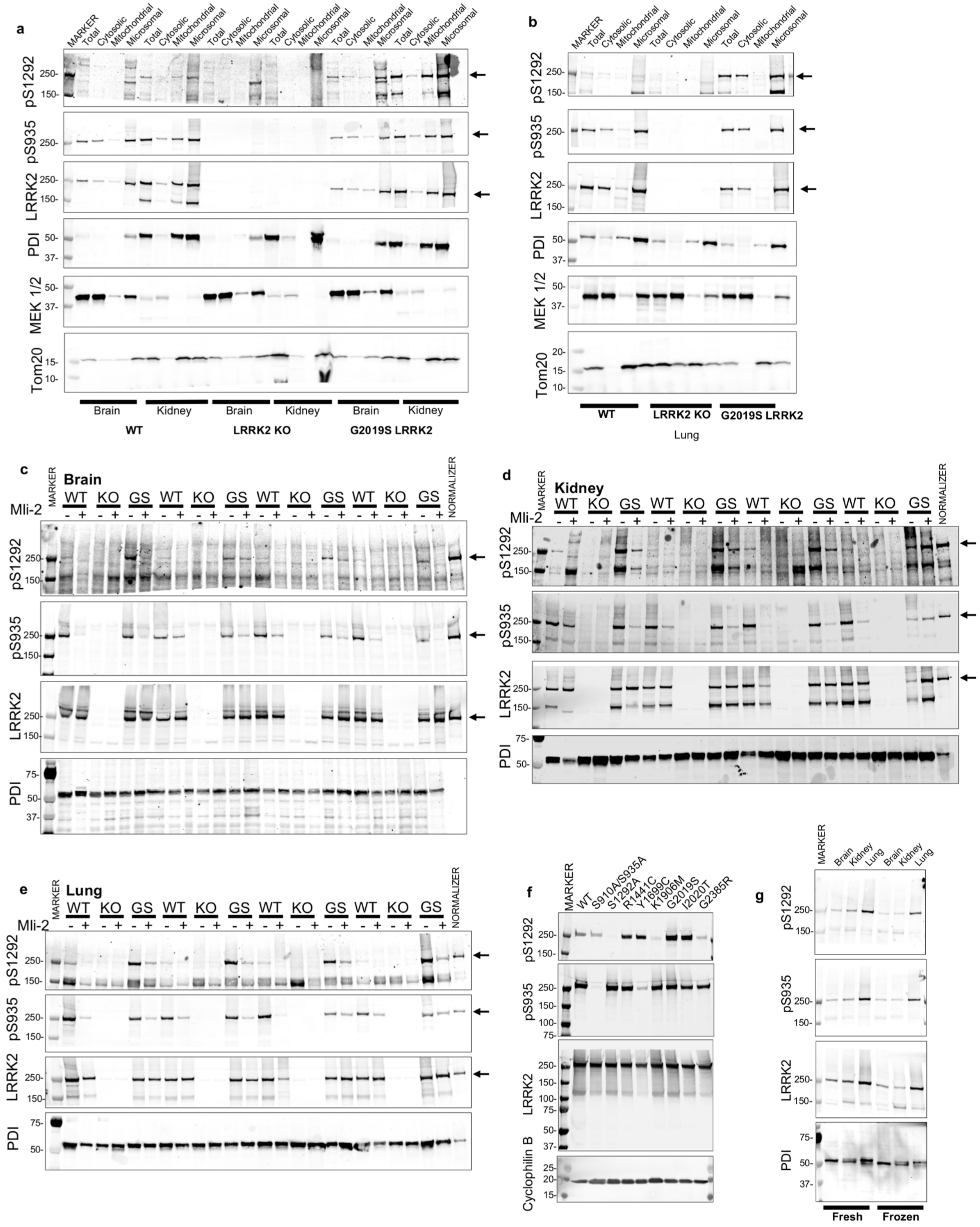

Supplementary Figure 1. Uncropped blots of figures 1 and 2 with molecular weight markers. (a, b) Uncropped blots of figure 2a, b; detection of total and phosphorylated LRRK2 in total, cytosolic, crude mitochondrial and crude microsomal fractions of mouse brain, kidney and lung. (c, d, e) Uncropped blots of figure 2c, f, i; detection of pS1292 and pS935 LRRK2 in brain, kidney and lung tissue from mice treated with MLi-2. (f) Uncropped blots of figure 1c; transiently expressed LRRK2 genetic variants probed for S1292 phosphorylation. (g) Uncropped blots of figure 2o; S1292 and S935 phosphorylation in fresh-processed and flash-frozen tissue from a G2019S LRRK2 mouse.
